# Society for Microbial Ecology, *Microbial Ecology in Health and Disease*, and the Future

**DOI:** 10.3402/mehd.v24i0.23315

**Published:** 2013-12-10

**Authors:** Tore Midtvedt

The Society for Microbial Ecology's (SOMED) 36th International Conference is now history. As stated by President Andy Onderdonk in the last SOMED newsletter, the meeting was a success. The Organizing Committee, headed by Professor Alojz Bomba, had done a marvellous job. The programme was well balanced with a many good lectures highly relevant for our society. We would, however, have liked to see even more participants and/or SOMED members, something that was discussed at the General Assembly meeting. The discussion resulted in a proposal from one of our councillors, Sandrine Claus, who a few days after the meeting came up with an excellent proposal:

**Figure:**
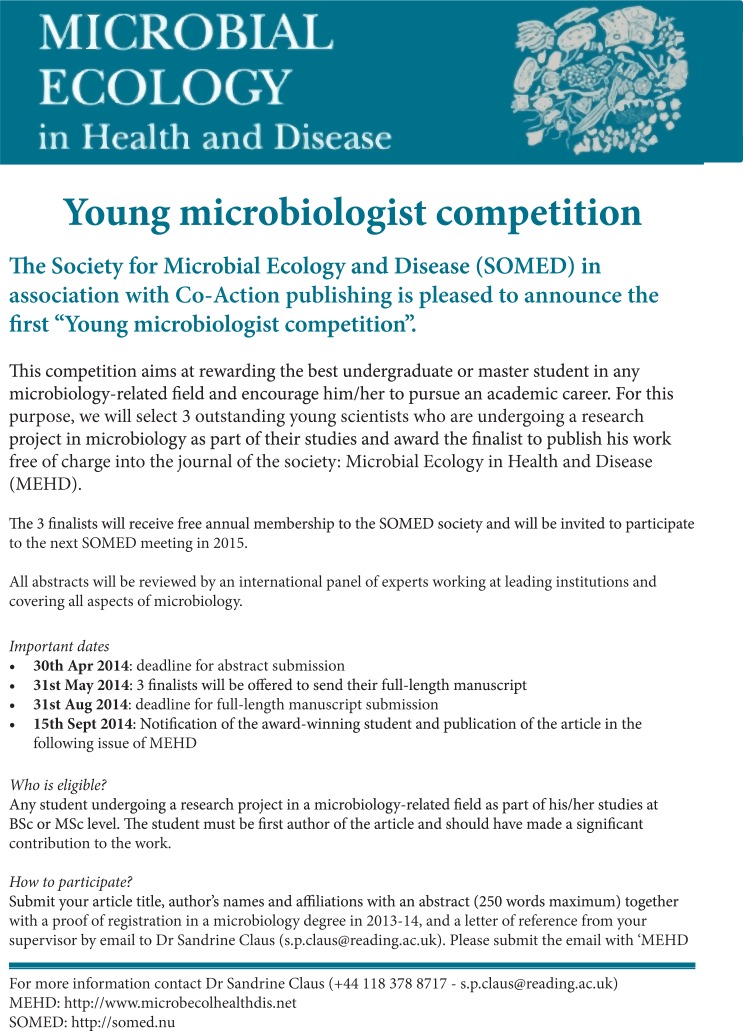


If they have not started already, I am quite sure that the SOMED Board will work hard to substantiate the excellent ideas that are outlined in Sandrine Claus’ proposal. I look forward to reading the winner's manuscript!

At the recent SOMED meeting in Kosice, Senior Publisher Anne Bindslev from Co-Action Publishing presented interesting information about *MEHD* and how the journal is developing. As the journal is now indexed in PubMed Central/PubMed, she encouraged all members of SOMED to support their society's own journal by submitting their best work to it, and also to have abstracts and/or proceedings from SOMED meetings published in *MEHD*.

At the meeting, four new members for the Editorial Board were recruited:

*N.V. Boyko*, Uzhhorod National University, Ukraine. Her last article in *Journal of the Science of Food and Agriculture* clearly demonstrates that some plant extracts can modulate platelet functions in humans.

*S.P. Claus*, University of Reading, UK. In *Cell Host Microbe*, she recently had an exciting article entitled “Fighting undernutrition: don't forget the bugs”!

*R.N. Fichorova*, Harvard Medical School, USA. Readers of *Microbial Ecology in Health and Disease* will already be familiar with her article in the 2013 volume: “Effect of feminine hygiene products on the vaginal mucosa.”

*K. Venema*, TNO, Netherlands. His recent article in *Best Practice & Research in Clinical Gastroenterology*, entitled “Experimental models of the gut microbiome,” is a “must” for young scientists entering that field.

These four names will now be added to the current editorial board: J. Artega, Columbia; A. Berstad, Norway; E. Bezirtzuglou, Greece; P. Carter, USA; P. Conway, Australia; M. Fons, France; W. Garrett, USA; E. Houpt, USA; K.A. Krogfelt, Denmark; V. Lazar, Romania; P. Mastrantoni, Italy; M. Mikelsaar, Estonia; I. Rowland, UK; and B. Shenderov, Russia.

Thus, the journal now has “ambassadors” all over the world, and in addition, they represent all the fields that are covered by *MEHD*. Several board members took up the challenge given in my Editorial from November 2012 and submitted excellent manuscripts for publication in *MEHD*. With the strengthened editorial board and an increasing number of readers around the world, in addition to PubMed indexing, I am confident that an increasing number of articles will be submitted to the journal from now on.

With a slight travesty of a statement given in the Editorial mentioned above, I will end by underlining:

Only together can we make 2014 the best year ever for SOMED and *MEHD*!

*Tore Midtvedt* Editor-in-Chief

